# The Relative Contributions of Different Wheat Leaves to the Grain Cadmium Accumulation

**DOI:** 10.3390/toxics10110637

**Published:** 2022-10-23

**Authors:** Chuang Ma, Lin Lin, Jun Yang, Hongzhong Zhang

**Affiliations:** 1 Henan Collaborative Innovation Center of Environmental Pollution Control and Ecological Restoration, Zhengzhou University of Light Industry, Zhengzhou 450001, China; 2 Institute of Geographical Sciences and Natural Resource Research, Chinese Academy of Sciences, Beijing 100101, China

**Keywords:** wheat, cadmium, grain, leaf, relative contribution

## Abstract

In the context of increasing atmospheric particles pollution, wheat cadmium (Cd) pollution caused by atmospheric deposition in agro-ecosystems has attracted increasing attention. However, the relative contribution of different wheat leaves-to-grain Cd accumulation is still unclear. We assessed the roles of different wheat leaves on grain Cd accumulation with field-comparative experiments during the filling stage. Results show that wheat leaves can direct uptake atmospheric Cd through stomata, and the flag leaf exhibited a higher Cd concentration compared to other leaves. The relative contribution of the leaves-to-grain Cd accumulation decreased gradually during the grain-filling period, from 34.44% reaching 14.48%, indicating that the early grain-filling period is the critical period for leaf Cd contributions. Moreover, the relative contribution of flag leaves (7.27%) to grain Cd accumulation was larger than that of the sum of other leaves (7.21%) at maturity. Therefore, the flag leaf is the key leaf involved in grain Cd accumulation, and controlling the transport of Cd from leaves to grains at the early filling period, particularly flag leaf, could help to ensure wheat grain safety, thus ensuring the safety of food production.

## 1. Introduction

Cadmium (Cd) contamination of wheat can pose serious threats to human health through bioaccumulation in the food chain [1,2]. It is not vital for life, having toxicity of 2–20 times higher than other metals; exposure to excessive amounts of Cd harms the human liver, testicles, kidneys, bones, cardiovascular system, and endocrine system [3]. Therefore, reducing the accumulation of Cd in wheat is crucial for preventing Cd-related health risks [4]. When considering a common soil Cd source, it is generally accepted that Cd accumulation in wheat grains is derived from the continuous absorption of soil Cd by the root system [5,6]. However, owing to the large-scale exploitation of natural resources and accelerated industrialization, problems related to atmospheric particles that contain heavy metal pollutants have become increasingly serious in agro-ecosystems [7,8]. It is estimated that the average annual Cd deposition flux on farmland in China is approximately 0.24–19.5 mg·m^−2^ [1,9]. Thus, more and more researchers have begun to observe the effect of Cd deposition on plant leaves [7,10,11]. For example, Cui et al. [12] found that atmospheric Cd was the predominant Cd source in the leaves of vegetable and maize (*Zea mays* L.) plants, even in Cd-contaminated soil. Sur et al. [3] reported that airborne Cd is an important factor that directly affects rice shoot uptake, and the contribution rate of atmospheric particles to Cd in rice grains reached 63.55% and 18.01% in moderately and severely polluted soils, respectively [11]. Our study also showed that the contribution rates of stored Cd reactivation in leaves at filling stage and atmospheric Cd newly absorbed by leaves at filling stage to Cd accumulation in grains were 19.76% and 11.97%, respectively, in a Cd-polluted farmland downwind of a lead-zinc smelter in Jiyuan city, China [10]. Thus, wheat leaves have an important effect on Cd accumulation in grains. However, to the best of our knowledge, the relative contribution of different wheat leaves to Cd accumulation in wheat grains is unclear. Especially for the vast majority of general farmland without soil Cd pollution in the main wheat-producing areas of the North China Plain where atmospheric Cd pollution is serious, further exploration of the relative contribution of leaves-to-grain Cd accumulation is important for controlling wheat grain Cd pollution.

In addition, as the main organ that absorbs Cd from the atmosphere, wheat leaves are also the main photosynthetic organ of the wheat plant and have substantial influences on grain formation and filling [7,13]. There are three primary functional leaves on the wheat stem during the filling period: the flag leaf, second leaf, and third leaf [13,14]. Of these, the flag leaf has the greatest impact on the wheat yield [15,16]. In contrast to other leaves, the flag leaf is located in the wheat canopy, which may make it easier to contact and absorb Cd in atmospheric particles [4,17]. More importantly, the re-transfer of Cd from the reservoir organs, including leaves, sheaths, and stems, to the grains is the main Cd accumulation pathway for the grains [4,18]. Wang et al. [19] found that Cd deposited on rice leaves was also re-activated and transported to the grains following re-orientation at the nodes during the filling period, which caused nearly half of the Cd absorbed by the plant to be concentrated in the grains. Re-orientation transport in nodes, particularly re-activation transport in flag leaves, was a major Cd accumulation pathway for rice grains [20]. However, Cd in different locations of wheat leaves needs to pass through a different number of nodes in the process of transport to the grain, and the nodes in the grass family have been shown to block Cd translocation from the shoots to the grains [2,5]. Therefore, compared with other leaves, flag leaves may absorb atmospheric cadmium more easily and transport it to grain efficiently [4,10], and furthermore, these differences lead to substantially different contributions from the three primary functional leaves to Cd accumulation in the grains.

In this study, we conducted a field experiment to evaluate the relative contributions of wheat leaves, particularly flag leaves, to grain Cd accumulation in areas subject to atmospheric Cd pollution. The objectives of this study were the following: (1) to quantify the relative contributions of different wheat leaves-to-grain Cd accumulation; (2) to investigate the critical period during which wheat leaves contribute to grain Cd accumulation. In short, the objective was to reveal the key leaves and the key period of leaf contribution to grain Cd accumulation under the condition of atmospheric Cd pollution, which provides a reference for controlling wheat grain Cd pollution.

## 2. Materials and Methods

### 2.1. Study Area and Experimental Design

The granary region of Henan Province, China, has a tremendous impact on China’s food supply. Xuchang is located in central Henan Province and receives plenty of sunshine and rainfall. The average annual temperature is 13–16 °C, with 671–736 mm of precipitation. The temperature, light, and precipitation parameters in this region provide a suitable environment for wheat growth. The test field used in this study was farmland near Xiaoji village in Xuchang city (Figure S1).

To understand the contributions of wheat leaves-to-grain Cd accumulation, experiments were conducted using defoliation treatments that removed the flag and other leaves from the wheat stems. Nine 3 m × 3 m plots were randomly selected and established as three experimental groups, each of which had three replicates. One group was established as a blank control group (CK). The first treatment involved the removal of only the flag leaves (FLR). The second treatment involved the removal of all leaves (ALR). All treatments were performed during the anthesis stage (AS; 27 April 2020). The leaves were removed from the wheat stems using stainless steel scissors. Standard wheat field cultivation methods were used in each experimental group. Then we determined the Cd concentrations in wheat tissues and grains, and this combined with SEM-EDS analysis results demonstrated the direct contribution of atmospheric Cd to the accumulation of Cd in leaves and grains. Finally, we quantified the relative contributions of flag leaves and other leaves-to-grain Cd accumulation at different grain filling stages.

### 2.2. Sampling Methods and Sample Pre-treatment

Wheat grain samples were collected on April 27 (anthesis stage, AS), 10 May (early grain filling stage, GS1), 19 May (mid-grain filling stage, GS2), and 31 May (grain maturity stage, MS) in 2020. Samples of approximately 50 wheat plants were collected randomly on each plot at a time. The collected wheat samples were subsequently subjected to chemical analysis in the laboratory. The samples were first rinsed with tap water to remove any obvious physical impurities from the surface. The samples were then immediately rinsed three times with ultrapure water and dried to a constant weight under the condition of 40 °C. Finally, they were marked and bagged until analysis.

Henan Province is the largest wheat-producing region in China; the soil is less contaminated by Cd, and according to the soil survey data of China, there is no Cd contaminated farmland in the whole study area (Xuchang City) [10,12]. Therefore, the study did not analyze the Cd concentration before wheat planting. Both soil and atmospheric particulate samples were collected simultaneously with the wheat samples. The soil samples were collected using the serpentine sampling method in each 3 m × 3 m sample plot. Three mixed samples were obtained each time and transported to the laboratory in sealed plastic bags. After air-drying, the samples were sieved before analysis [4,21]. Atmospheric particulate samples were collected using glass samplers (15 cm in diameter and 30 cm in height), which were fixed approximately 4 m above the ground on a pole near the wheat to prevent secondary contamination. An appropriate amount of hexylene glycol was added to each sample to maintain moisture in the sampling medium. Impurities and sediment were removed during sampling by repeated rinsing with ultrapure water. Sample mixtures were stored in a refrigerator until analysis [22]. The PM_2.5_ and PM_10_ monitoring data were obtained from the China National Environmental Monitoring Centre and the local Environmental Protection Bureau (http://www.cnemc.cn/sssj/ (accessed on 25 March 2022)).

### 2.3. Analytical Methods

1. Physico-chemical analyzes of the samples

Plant samples were placed in a triangular flask; digested with 8 mL HNO_3_, 2 mL HClO_4_, and 2 mL H_2_O_2_; covered and soaked overnight; and digested with heating on an electric hot plate. The soil and atmospheric deposition samples were then digested with 36–38% HCl, 65–68% HNO_3_, 40% HF, and 70–72% HClO_4_ (w%). The entire process was conducted on an electric hot plate [4]. To extract and analyze the different forms of Cd from the soil and atmospheric samples, a continuous extraction method adapted from the Reference Bureau of the European Communities Commission was utilized. (The specific method is given in Table S1.) In addition, for quality control, nationally recognized soil and plant reference materials (GBW07454 and GBW10046, respectively) were utilized as controls, with Cd recoveries of 90–105% and 93–107%, respectively. All samples were analyzed in duplicate. Cd contents were determined using atomic absorption spectrometry (ZEENIT-700P analyzer, Jena, Germany). All reagents used in the experiments were of high purity.

The soil organic matter was measured using the Walkley-Black dichromate oxidation method [23]. Soil pH was determined using a 1:2.5 (*w*:*v*) soil-to-water ratio [1]. The samples were dried in an oven at 40 °C for 96 h, ground and sieved with 200 mesh to obtain the total nitrogen and total phosphorus, and passed through a 0.85-mm sieve to obtain the available potassium [12], the contents of which were determined by an automatic elemental analyzer (VARIO EL III, Elemental Analysensysteme GmbH, Hanau, Germany) [4].

2. Plant morphology

The adaxial stomatal morphology of the aboveground tissues of the wheat plants during the maturity stage was observed using scanning electron microscopy and energy dispersive X-ray spectroscopy (SEM-EDS) (JSM-6490LV, JEOL Ltd., Tokyo, Japan; Oxford, INCA X-sight, UK). The leaves were freeze-dried in a vacuum freeze-dryer (Beijing Boyikang Experimental Instruments Co., Ltd., Beijing, China), then immediately placed on prepared copper bases affixed with a conductive adhesive, and sprayed with platinum for 160 s to improve its conductivity and secondary electron yield, and thus clearer microstructure of leaves were obtained. Finally, SEM-EDS was used to analyzing the pores and particles on the blade. The weights and atomic percentages of Cd and other elements were obtained by EDS analyses of the stomata and the particulate matter observed in the SEM images.

### 2.4. Grain Filling Rate and Grain Cd Accumulation Rate Calculation

The grain-filling rate and grain-Cd-accumulation rate were calculated using the following equations [4]:

(1) $$H_{1}\;=\;\frac{\Delta m}{\Delta t}$$

(2) $$H\;=\;\frac{\Delta n}{\Delta t},\;n\;=\;m\;\times \;c$$

where *H*_1_ represents the grain filling rate (mg·1000 grains^−1^·d^−1^), *m* represents the thousand-grain weight of wheat during each growth period, ∆*m* represents the increase in the thousand-grain weight during two adjacent periods (mg), *H* represents the grain Cd accumulation rate (mg·1000 grains^−1^·d^−1^), *c* represents the grain Cd concentration during each growth period (mg·kg^−1^), *n* represents the Cd accumulation per thousand grains during each period (mg), and ∆*n* represents the increase in Cd accumulation during two adjacent periods (days).

### 2.5. Statistical Analyses

All independent sample data were subjected to the Shapiro-Wilk normality and variance homogeneity tests. The significance of the differences was determined using one-way analysis of variance (ANOVA) or independent sample *t*-tests. The Kruskal-Wallis non-parametric test was also used to investigate the significance of differences in sample data that did not pass the one-way ANOVA test. All analyses were conducted using the IBM SPSS Statistics software package (version 25, SPSS Inc. Chicago, IL, USA), and figures were created with Origin 9.0.

## 3. Results and Discussion

### 3.1. Regional Atmospheric Particulate Matter and Soil Cd Pollution

Henan Province is the largest wheat producing region in China, and the soil is less contaminated by heavy metals. In the study area, the soil texture was loam, and the soil type was calcaric fluvisol. Soil properties of the study area were as follows: the organic matter content was 11.6 g·kg^−1^; the pH was 7.9; the total contents of nitrogen and phosphorus were 0.87 g·kg^−1^ and 0.55 g·kg^−1^, respectively; and the available potassium was 115.56 g·kg^−1^. The soil Cd concentration of the study area was 0.25 mg·kg^−1^, which is substantially lower than the Soil Environment Quality Risk Control Standard for Soil Contamination of Agriculture Land (GB 15618–2018) of 0.6 mg·kg^−1^ (pH > 7.0). This indicates that the soil in the study area was not contaminated.

**Figure 1:**
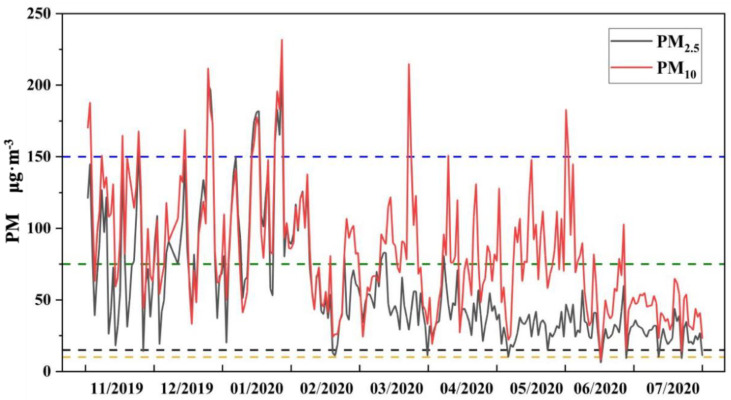
Daily average mass concentration of PM_2.5_ and PM_10_ in study area.

However, due to industrial production pollution and automobile exhaust, especially in winter, as well as coal-fired heating, air pollution is frequent during the wheat-growing period [24,25]. Figure 1 shows the daily variations in PM_2.5_ and PM_10_ in Xuchang city during the entire wheat−growing period (From November to July of the next year). According to the growth stage of wheat, late April is the anthesis stage, early May is the early grain filling stage, middle May is the mid-grain filling stage, and the end of May is the grain maturity stage. According to the World Health Organization Standard (10 μg·m^−3^) and the US National Ambient Air Quality Standard (15 μg·m^−3^) [26,27], the atmospheric particulates were in a serious polluted state during the entire wheat-growing period, and the pollution was particularly obvious during the winter, even with reference to the more lenient Chinese standard of 75 μg·m^−3^ [28,29]. From February to April, pollution and the overall PM_2.5_ and PM_10_ concentrations decreased, and from May to July, less polluted weather occurred, which is consistent with particulate matter trends observed in other areas of the North China Plain [24,25]. Studies have shown that heavy metals are mainly found in fine particulate matter, such as PM_2.5_ [4,30]; the mean PM_2.5_ and PM_10_ values in the study area were calculated as 58.96 and 83.11 μg·m^−3^, respectively. The PM_2.5_ accounted for 70% of the PM_10_, which is consistent with the 60–80% average ratios in most developed and developing countries [29].

To further explore the pollution risk of atmospheric particulates, atmospheric particulates were collected during the filling period, and their Cd contents and morphological distribution were determined. The results indicate that the atmospheric particulate Cd concentration showed large temporal and spatial variations [3], fluctuating between 2–4 mg·kg^−1^, which was 8–16 times higher than the soil Cd concentrations in the study area (Table 1). More importantly, heavy metal hazards depend on both their concentrations and morphological distributions [31,32]. Table 1 shows that the morphological distributions of Cd differed between the soil and atmospheric particulate matter. The acid soluble, reducible, oxidizable, and residual states of the soil Cd were 15.22% ± 1.35%, 20.83% ± 0.66%, 22.34% ± 1.01%, and 41.60% ± 2.75%, respectively. However, the atmospheric Cd acid soluble, reducible, oxidizable, and residual states were 24.70% ± 1.68%, 16.63% ± 1.96%, 17.56% ± 1.93%, and 41.11% ± 5.05%, respectively. The acid soluble state of atmospheric deposit Cd was significantly higher than that of the soil, indicating that its biological activity was also higher than that of the soil (*p <* 0.05). In effect, the high concentrations and activity of atmospheric Cd provided a rich source for the wheat leaves [18,33]. The study area represents typical atmospheric Cd pollution on the North China Plain.

### 3.2. Mechanisms of Cd Uptake in Leaves Based on SEM-EDS

**Figure 2:**
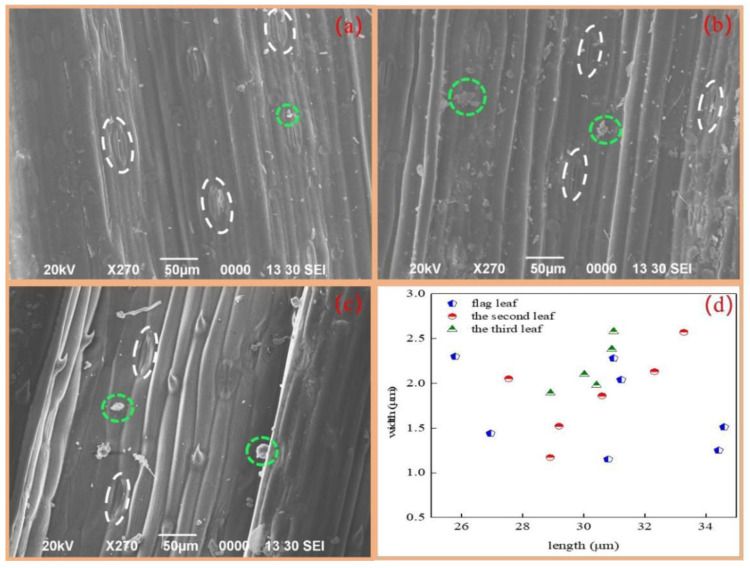
Stomatal distribution of wheat flag leaf (**a**), the second leaf (**b**), the third leaf (**c**), and stomatal pore size map (**d**) of wheat tissues. The magnification is 270×. Inside the white solid line frame are stomata. Inside the green dotted frame are the atmospheric deposition particulates.

Heavy metals in the atmosphere can penetrate through adsorption and can be internalized in the cuticle through the stomata [34,35]. Previous studies have shown that the absorption of atmospheric particles by stomata is the most convenient uptake pathway [36,37]. Therefore, in order to investigate the uptake pathway and mechanism of atmospheric Cd in wheat leaves, we used SEM-EDS to analyze the stomatal distributions of the flag, second, and third leaves. As can be seen from Figure 2, there were many stomata distributed on the leaves, and the stomatal size was approximately 25–35 μm in length and 1–2.8 μm in width, which is considerably larger than the size of PM_2.5_ and indicates that leaves provide a convenient pathway for the absorption of atmospheric Cd in PM_2.5_ [7,30].

**Figure 3:**
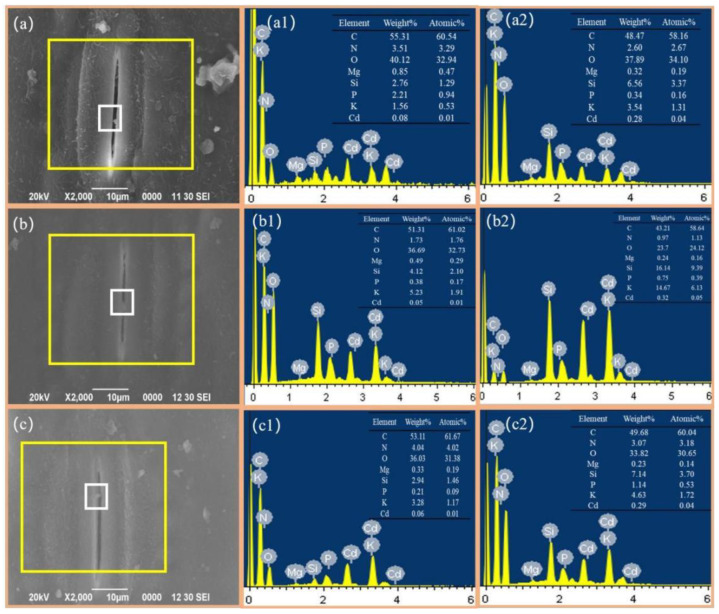
EDS analysis of stomatal surface (**a**) flag leaf, (**b**) the second leaf, (**c**) the third leaf. The scanning area (**a1**,**b1**,**c1**) is shown by a yellow wire frame. The scanning area (**a2**,**b2**,**c2**) is shown by a white wire frame. The magnification of leaves is 1000×, the magnification of husk and awn is 1000×.

In addition, we found many granules distributed on the leaf surfaces (Figure 2) that could be absorbed into the plant leaves through the stomata and cuticle channels [38] or diffuse into the leaves through surface waxes [36,39]. The ability of the leaf stomata to absorb atmospheric Cd was confirmed by the EDS analysis (solid yellow box in Figure 3a), which yielded an atomic Cd percentage of 0.08% (Figure 3(a1)). The region around the stomata (white solid box in Figure 3(a2)) exhibited a 0.20% increase in the atomic Cd percentage to 0.28% (Figure 3(a2)), which indicates that the wheat leaves could directly impact the uptake of atmospheric fine particulate matter Cd through stomata, which might be responsible for grain Cd accumulation under atmospheric particles pollution [14,30,40].

### 3.3. Cd Concentrations in Wheat Tissues and Grains

**Figure 4:**
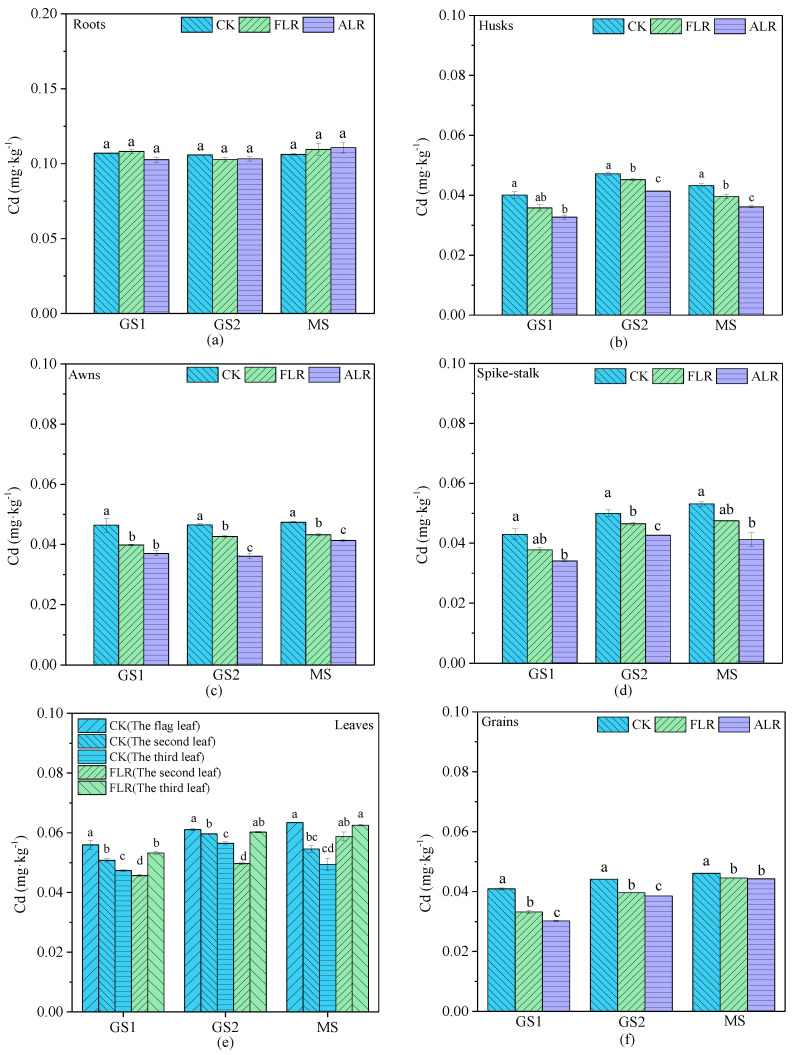
Cd concentration in wheat tissues in different grain filling stages. Note: Cd concentrations in roots (**a**), husks (**b**), awns (**c**), spike-stalk (**d**), leaves (**e**) and grains (**f**) under different treatments located at different stages. Different letters (a, b, c, d) indicate that there are significant differences in the same stage (*p* < 0.05). The data are expressed as the means ± SD (n = 3); GS1 = early grain filling stage, GS2 = middle grain filling stage, MS = mature stage.

The Cd concentration of wheat roots was 0.1–0.15 mg·kg^−1^, which is substantially higher than those of the leaves and spikes (husks, spike−stalks, and awns) (Figure 4). This is consistent with the findings of Zhang et al. [2] in eight Chinese wheat cultivars. The Cd concentrations of the roots did not demonstrate pronounced differences among the three treatments (CK, FLR, and ALR), likely because Cd in the root system originates primarily from the soil, and the Cd absorbed by the above-ground was rarely transferred to the root system [12,18,41].

It is notable that the Cd concentrations of the leaves decreased from the top of the plant to the bottom, as the highest concentration was in the flag leaf, followed by the second and third leaves (Figure 4e). However, previous studies have generally concluded that Cd concentrations in plants exhibit gradual decreases from the bottom to the top of the plant, owing to a gradual reduction in heavy metal availability [16,42]. This may be due to the relatively uncontaminated soil in the study area, where atmospheric Cd was the main source of pollutant uptake by the above-ground parts. Waxes on the wheat leaf surfaces trap dust particles containing heavy metals, which are then absorbed into the leaves [4,17]. The flag leaves located in the wheat canopy are most likely to absorb atmospheric Cd and block it from leaves located lower on the plant [4,43,44]. Therefore, atmospheric Cd is more readily absorbed by the flag leaves and transported to the grains. Also, it should be noted that the dry matter mass of the flag leaf, as the leaf that contributes most to the yield, is significantly larger than that of the second and third leaves [14]. Thus, more Cd uptake by the flag leaf can provide a larger source of Cd to the grains. Therefore, the accumulation of Cd in leaves at different locations on the plants differed.

The Cd concentration of the spikes (husks, spike-stalks, awns) and grains ranged from 0.03 to 0.05 mg·kg^−1^, increasing as grain filling advanced (Figure 4). The Cd concentrations of the grains increased gradually, although the differences between the three treatments decreased gradually as grain maturation progressed (Figure 4f). The Cd concentrations of the grains under the CK treatment were significantly (*p <* 0.05) higher than those under the FLR and ALR treatments at maturity, with no significant difference between the FLR and ALR treatments (Figure 4f). These results indicate that wheat-leaves Cd could transfer to the grain at the filling stage, and leaf removal could effectively reduce the Cd concentrations of the grains, as the FLR and ALR treatments achieved similar results. This may be due to the fact that Cd in the leaves is transferred to the grains with the filling material, and the contribution of flag leaves to the grain yield is significantly larger than those of the other leaves [16,45]. Therefore, we investigated the rate of grain filling and Cd accumulation in wheat.

### 3.4. Grain Cd Accumulation Characteristics during the Filling Stage

**Figure 5:**
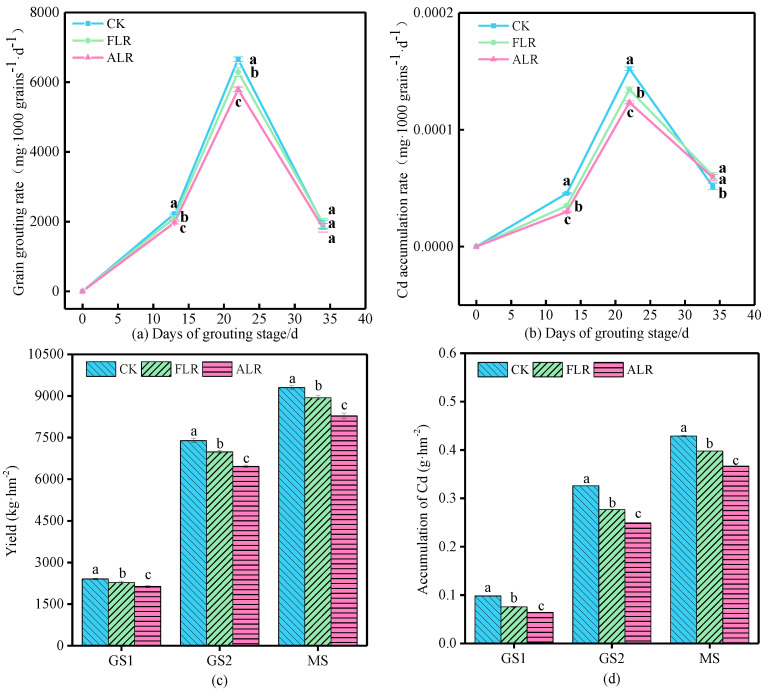
Grain filling rate (**a**), Cd accumulation rate (**b**), wheat yield (**c**), and accumulation of grain Cd (**d**). Note: Different letters (a, b, c) indicate significant differences (*p* < 0.05) between CK, FLR, and ALR in the same stage.

Despite the removal of leaves in the ALR and FLR treatments, the wheat spikes and other organs still performed photosynthesis [16,46]; wheat yields and grain Cd accumulations from the three treatments exhibited a gradually increasing trend (Figure 5). Compared with the yield and Cd accumulation in the CK treatment, both the yield and Cd accumulation decreased substantially in the FLR and ALR treatments (Figure 5). Further, the yield and grain Cd accumulation were substantially higher in the FLR treatment than in the ALR treatment. This indicates that leaf removal reduced Cd accumulation in the grains [4,47].

The grain-Cd-accumulation rate and grain-filling rate patterns were consistent among the three treatments; each increased to a maximum at 25 days, followed by a decrease (Figure 5b), which indicate that the timing of Cd accumulation in grain was positively correlated with grain biomass accumulation [15]. The Cd accumulation and grain filling rates of the ALR and FLR treatments were substantially lower than those of the CK treatment. The ALR treatment yielded substantially lower rates than the FLR treatment (Figure 5a,b). Therefore, leaf removal could reduce the concentrations of accumulated Cd and the accumulation rate in wheat grains considerably, which indicates that flag leaves and other leaves both have substantial impacts on grain Cd accumulation and are the source for Cd in the grains.

### 3.5. Relative Contributions of Wheat Leaves-to-Grain Cd Accumulation

**Figure 6:**
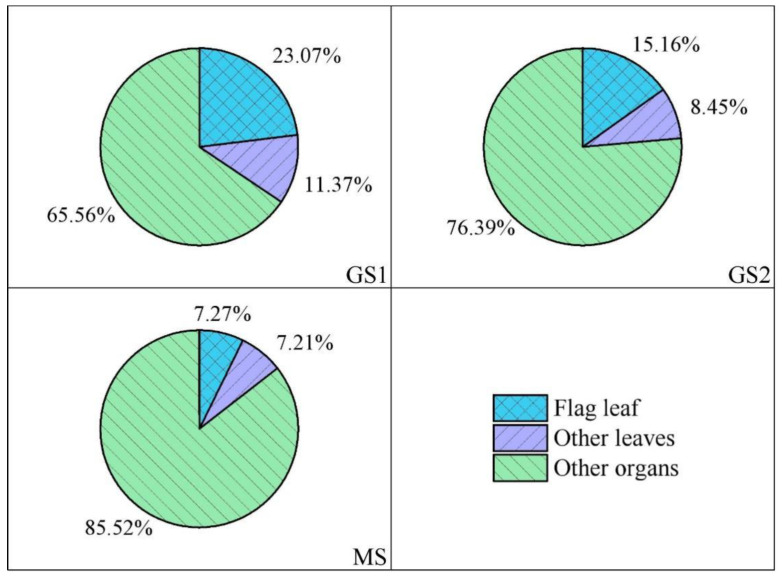
Relative contribution of wheat leaves-to-grain Cd. Note: GS1 = early grain filling stage, GS2 = middle grain filling stage, MS = mature stage.

By subtracting the accumulated Cd in grains of the CK treatment from those of the FLR and ALR groups, then dividing the result by the Cd accumulated under the CK treatment, we found that the contribution of the flag leaf to grain Cd decreased from 23.07% in GS1 to 7.27% at maturity. The results of this calculation for the other leaves decreased from 11.37% to 7.21% over the same period (Figure 6). This indicates that the relative contributions of each leaf type (flag and other leaves) to grain Cd accumulation decreased. Previous studies have shown that wheat leaves gradually senesce during the filling process, while the contributions from other non-leaf organs (such as spikes and stems) to the yield increase gradually [48,49]. In this study, the rate of grain Cd accumulation was consistent with the grain filling rate (Figure 5a,b), which then led to Cd accumulation in the leaves that was transferred to the grains during the early grain filling period. Thus, the contribution of the leaves-to-grain Cd decreased gradually and the early grain filling was the key period of leaf contribution to grain Cd accumulation.

Furthermore, the relative contribution of the flag leaf to grain Cd was larger than those of the sum of other leaves (Figure 6), thus, the flag leaf is the key leaf for grain Cd accumulation. It is mainly because of the flag leaf is the most important photosynthetic organ that promotes grain filling [16,45], and is located in the wheat canopy, allowing it to absorb atmospheric Cd more easily [20,47] and accumulate substantially more Cd than the other leaves (Figure 4e). In addition, the relatively higher contribution of flag leaves may be related to the different Cd transport pathways from different leaves to the wheat grains, different wheat leaves Cd have to pass through different numbers of nodes during Cd transport to the grains [20,50]. The node is a necessary pathway for the final translocation of Cd to grains and determines the process of transfer and distribution of metal elements (such as Zn, Cd, etc.) [2,20,51,52]. According to recent studies, nodes in the grass family have been shown to block Cd translocation from the shoots to the grains [2,5], which may lead to different efficiency of Cd transport from different leaves to grains. The flag leaf Cd has the shortest transport distance and the least number of nodes than other leaves [4,17], and further lead to substantially different contributions of the three primary functional leaves to Cd accumulation in the grains. In summary, these results demonstrate differences in the atmospheric Cd uptake and transport pathways by leaves at different locations, resulted in the flag leaf contributing more to grain Cd accumulation than all other leaves.

### 3.6. Future Applications of Research

Through this study, we found that the flag leaf is the key organ for Cd pollution in wheat grains, while the contribution of leaves-to-grain Cd accumulation mainly occurred in the early grain filling period, which provided a target organ and a key period for prevention and control of atmospheric Cd pollution. Therefore, for the control of wheat grain Cd pollution, the first consideration is to reduce the absorption of atmospheric Cd by the flag leaf and to inhibit the Cd transport from the flag leaf to grains in the early grain-filling period. For example, Yang et al. [47] inhibited the transfer of Cd from flag leaves to spikes and grains by foliar spraying of 2,3-dimercaptosuccinic acid (DMSA), which caused a 47.95% decrease in Cd concentration in rice grains. Meanwhile, Yang et al. [20] discovered that the transport of Cd from the flag leaf and internodes to grains would be blocked by spraying glycerol on the foliage during the filling period, which reduced Cd accumulation in the grains by 60.4%. However, this study did not explore the effect of nodes on Cd transport in wheat leaves, and the current studies on plant nodes were focused on root uptake and the transport of soil Cd [2,20,51,52]. Therefore, the role of different nodes for atmospheric Cd transport in wheat leaves, especially whether similar patterns exist for the transport of atmospheric Cd in wheat nodes, needs to be further clarified to provide an effective way for efficient "flow control" regulation of Cd accumulation in grains.

## 4. Conclusions

Wheat leaves can accumulate considerable amounts of Cd through stomata directly absorbing atmospheric Cd, even in uncontaminated soil. The flag leaf exhibited a higher Cd concentration compared to other leaves, and the contribution of the leaves-to-grain Cd accumulation decreased gradually, reaching 14.48% at maturity. Among them, the contribution of flag leaves (7.27%) to grain Cd accumulation was larger than that of all other leaves (7.21%). Therefore, the flag leaf is a critical leaf organ for Cd accumulation in wheat grains, and the early grain-filling period was the crucial period for leaf Cd contributions. These results demonstrate the need for preventing the transport of Cd from leaves, particularly flag leaves, to wheat grains at the early filling period to control Cd contamination in wheat grains.

## Figures and Tables

**Table 1:** Cd concentration and chemical state distribution of Cd in the soil and atmospheric particles.

Environmental Medium	Cd (mg·kg^−1^)	Acid Soluble State	Reducible State	Oxidizable State	Residual State
Soil	0.25 ± 0.04	15.22% ± 1.35%	20.83% ± 0.66%	22.34% ± 1.01%	41.60% ± 2.75%
Atmospheric particles	3.1 ± 0.25	24.70% ± 1.68%	16.63% ± 1.96%	17.56% ± 1.93%	41.11% ± 5.05%

## Data Availability

Not applicable.

## Supplementary Materials

The following supporting information can be downloaded at: https://www.mdpi.com/article/10.3390/toxics10110637/s1; Figure S1: Study area; Table S1: Continuous extraction method of BCR for soil, atmospheric particles.
