# Supplementary material for: The Relative Contributions of Different Wheat Leaves to the Grain Cadmium Accumulation
Source: Toxics. 2022 Oct 23;10(11):637. doi: 10.3390/toxics10110637 (PMC9697351; doi:10.3390/toxics10110637)
Supplement: Supplementary file 1 [file toxics-10-00637-s001.zip › toxics-1983388-supplementary.pdf]

The Relative Contributions of Different Wheat Leaves to the Grain Cadmium Accumulation

Chuang Ma [1], Lin Lin [1], Jun Yang [2,*] and Hongzhong Zhang [1,*]

[1] Henan Collaborative Innovation Center of Environmental Pollution Control and Ecological Restoration, Zhengzhou University of Light Industry, Zhengzhou 45000, China
[2] Institute of Geographical Sciences and Natural Resource Research, Chinese Academy of Sciences, Beijing 100101, China

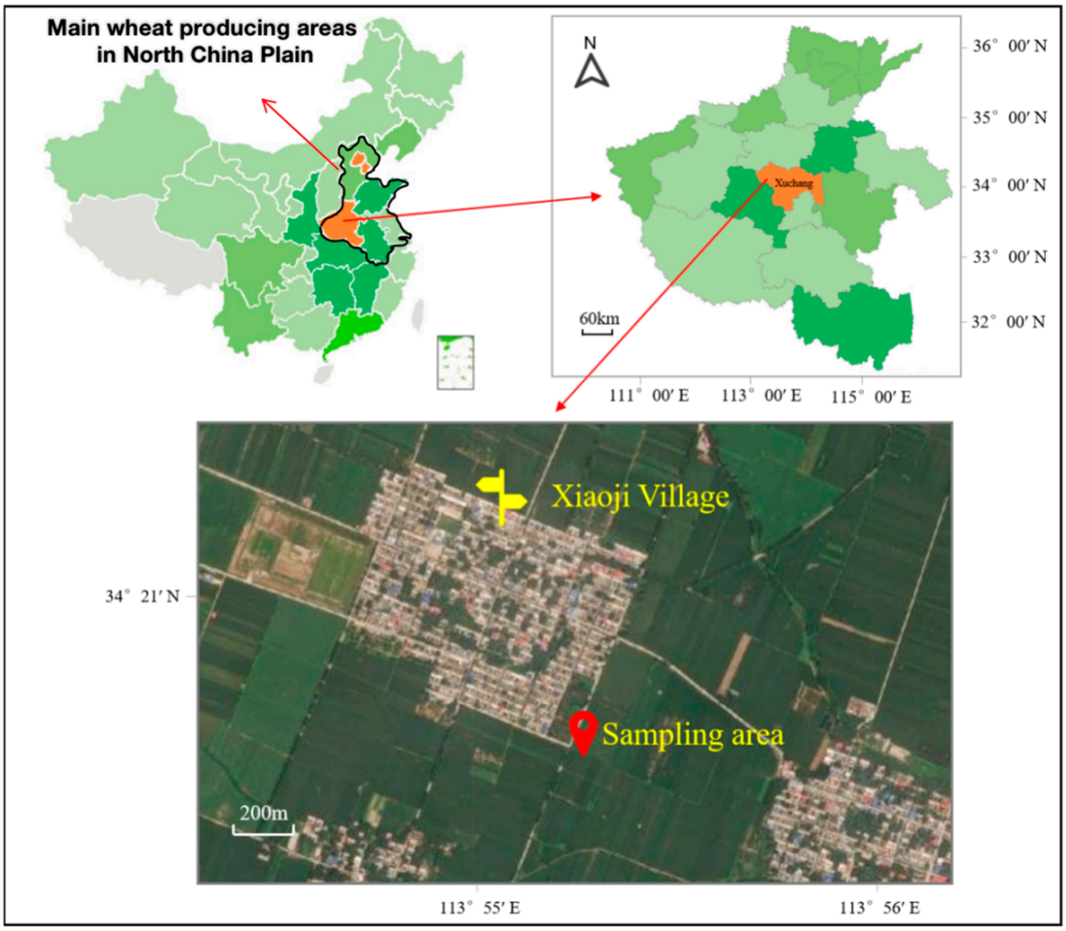

Figure S1. Study area.

Table S1. Continuous extraction method of BCR for soil, atmospheric particles.

Fraction	Reagent	Procedure
Acid soluble fraction	0.1 mol·L $^{-1}$ CH_3 COOH(AR)	Accurately taking 0.500 0 g sample in a 40 mL centrifuge tube, add 20 mL 0.1 mol·L $^{-1}$ CH_3COOH, shake at 160 r·min $^{-1}$ for 16 h at room temperature, centrifuge at 3 000 r·min $^{-1}$ for 20 min, and take the supernatant to be tested
Reducible fraction	0.5mol·L $^{-1}$ $NH_4OH·HCl$(AR) (pH=1.5)	The first step residue was added 0.5 mol·L $^{-1}$ $NH_4OH·HCl$ and shaken at (160±5) r·min $^{-1}$ for 16 h at room temperature, centrifuged at 3 000 r·min $^{-1}$ for 20 min, and the supernatant was sampled
Oxidizable fraction	30%H_2O_2 (GR)1mol·L $^{-1}$$NH_4OAc$ (AR)(pH=1.5)	Add 5 mL of 30% H_2O_2 to the residue of the second step, shake well, and after standing for 1 h, heat the water bath (85±2) ℃ to nearly dry H_2O_2, cool, and then add 5 mL of 30% H_2O_2 in a water bath until nearly dry. After cooling, add 25 mL of 1 mol·L $^{-1}$ NH_4OAc and shake at (160±5) r·min $^{-1}$ for 16 h at room temperature. Centrifuge at 3 000 r·min $^{-1}$ for 20 min and take the supernatant to be tested
Residual fraction	HNO_3(GR):HCl(GR):HF(GR):$HClO_4$ (GR) = 6:2:1:0.5	Accurately weigh approximately 0.1000 g of the sample (soil or atmospheric dust) in a Teflon digestion tank and use a mixed acid system (HNO_3 : HCl : HF=6 : 2: 1) for microwave digestion. In the digestion, each sample was made in two parallel, blank control at the same time, and the national standard substance (GBW07454) was added for quality control. The determination was performed using an atomic absorption spectrophotometer.
